# Supplementary material for: Splice-Junction-Based Mapping of Alternative Isoforms in the Human Proteome
Source: Cell Rep. Author manuscript; Available in PMC 2020 Jan 15. (PMC6961840; doi:10.1016/j.celrep.2019.11.026)
Supplement: 2 [file NIHMS1546469-supplement-2.zip › DF1/PXD009737/TestisLysC/TestisLysC_31_PRDX5_AAAAMAPIKTHLPGFVEQAEALK.pdf]

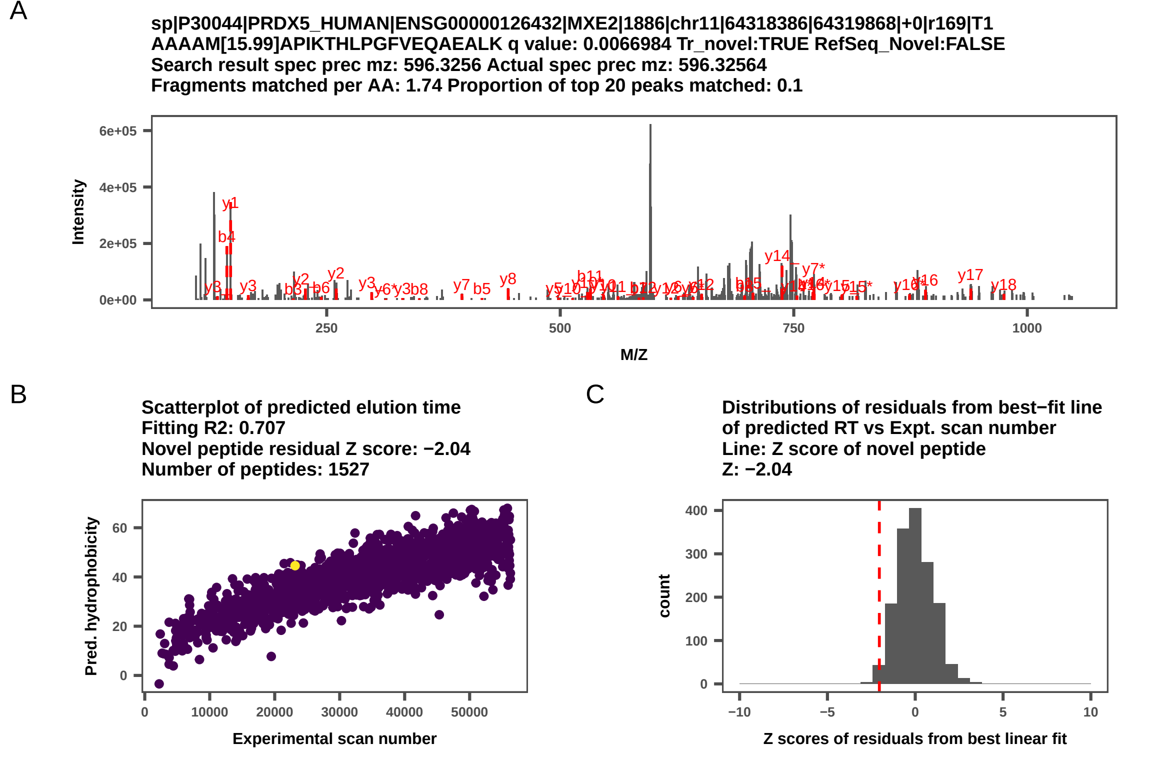
A
sp|P30044|PRDX5_HUMAN|ENSG00000126432|MXE2|1886|chr11|64318386|64319868|+0|r169|T1
AAAAM[15.99]APIKTHLPGFVEQAEALK q value: 0.0066984 Tr_novel:TRUE RefSeq_Novel:FALSE
Search result spec prec mz: 596.3256 Actual spec prec mz: 596.32564
Fragments matched per AA: 1.74 Proportion of top 20 peaks matched: 0.1
Intensity
6e+05
4e+05
2e+05
0e+00
250
500
750
1000
M/Z
y1
b4
y3
y2
y7
b5
y8
b11
y14
y7*
y16
y17
y18
B
Scatterplot of predicted elution time
Fitting R2: 0.707
Novel peptide residual Z score: −2.04
Number of peptides: 1527
Pred. hydrophobicity
60
40
20
0
10000
20000
30000
40000
50000
Experimental scan number
C
Distributions of residuals from best−fit line
of predicted RT vs Expt. scan number
Line: Z score of novel peptide
Z: −2.04
count
400
300
200
100
0
−10
−5
0
5
10
Z scores of residuals from best linear fit
